# Modulation of specific inhibitory networks in fatigued locomotor muscles of healthy males

**DOI:** 10.1007/s00221-017-5142-x

**Published:** 2017-12-06

**Authors:** Stuart Goodall, Glyn Howatson, Kevin Thomas

**Affiliations:** 1 Department of Sport, Exercise, and Rehabilitation, Faculty of Health and Life Sciences, Northumbria University, Newcastle-upon-Tyne, NE1 8ST UK; 2 Water Research Group, School of Environmental Sciences and Development, Northwest University, Potchefstroom, South Africa

**Keywords:** Brain, Contraction, Maximal, Muscle, Sustained

## Abstract

Reduced maximal force capability of skeletal muscle, as a consequence of exercise, can be due to peripheral or central fatigue mechanisms. In upper-limb muscles, neuromuscular fatigue is concurrent with reduced corticospinal excitability and increased inhibition (lengthened corticospinal silent period [CSP]; reduced short-interval intracortical inhibition [SICI] ratio). However, it is unclear whether these adjustments occur in response to fatiguing exercise of locomotor muscles. This study examined the effect of fatiguing, maximal, knee-extensor exercise on motor cortical excitability and inhibition. Thirteen males performed three 30-s maximal, isometric contractions with the dominant knee-extensors (MVC1, MVC2 and MVC3), separated by 60 s. At the end of, and between each MVC, neuromuscular fatigue, corticospinal excitability, CSP and SICI were assessed with supramaximal stimulation of the femoral nerve, and motor cortical stimulation, respectively. Repeated MVCs caused progressive reductions in MVC (− 10, − 24 and − 29%, respectively, *P* ≤ 0.01), along with significant peripheral (reductions in potentiated twitch of − 23, −53 and − 60%, respectively, *P* < 0.001) and central (reductions in VA of − 10% and − 13% post-MVC2 and 3, respectively, *P* ≤ 0.01) fatigue. Following MVC1 corticospinal excitability was reduced, and remained depressed thereafter. CSP increased in duration and remained longer throughout the protocol; whereas, no change in SICI was observed. Repeated, sustained, maximal contractions of the knee-extensors elicited substantial peripheral and central fatigue that was accompanied by a concomitant reduction in corticospinal excitability. However, divergent responses exist between inhibitory networks within the motor cortex, the activity of inhibitory networks mediated by GABA_B_ are increased, whereas those mediated by GABA_A_ are not.

**Electronic supplementary material:**

The online version of this article (10.1007/s00221-017-5142-x) contains supplementary material, which is available to authorized users.

## Introduction

Fatigue is a universal phenomenon characterised by sensations of tiredness and weakness during or following exertion, which is underpinned and/or modulated by multiple physiological and psychological processes. Exercise, and the consequent disruption to homeostasis, is a particularly potent stimulus to elicit fatigue, the mediators of which will vary depending on the exercise task. For exhaustive exercise of a single muscle group, fatigue is accompanied with a reduction in voluntary force (termed “muscle fatigue”; Gandevia 2001) that is primarily underpinned by adjustments in central nervous system (CNS) and muscle function. Peripheral mechanisms of fatigue refer to adjustments in contractile function, whereas central mechanisms of fatigue refer to an inability to voluntarily activate the involved muscles. Peripheral and central mechanisms of fatigue, and their contributions to the force loss experienced after exhaustive single limb exercise, have been well-studied (Carroll et al. 2017). More recently the CNS response to exhaustive exercise, in particular the excitability of motor cortical networks, have been increasingly studied (Tergau et al. 2000; Benwell et al. 2006; Maruyama et al. 2006; Takahashi et al. 2009; Hunter et al. 2016), but how these networks respond to lower-limb muscle fatigue is unknown.

Excitatory and inhibitory responses within the motor cortex can be studied using transcranial magnetic stimulation (TMS). When TMS is delivered over the motor cortex during a voluntary contraction, a motor evoked potential (MEP) is generated in the target muscle. As noted by many others, MEPs evoked by a standard stimulus in a resting muscle reduce following a fatiguing contraction (Brasil-Neto et al. 1993; Samii et al. 1996; Maruyama et al. 2006). When such a response in the MEP is observed, and responses to peripheral nerve stimulation are unchanged, it can be concluded that corticospinal excitability is reduced (Kotan et al. 2015). Spinal excitability is known to reduce with fatigue (McNeil et al. 2009) and this has been considered as a mechanism involved in the reduced MEP amplitude. Other possible explanations for this reduction are the change in postsynaptic properties of cortical neurons, neurotransmitter depletion (Brasil-Neto et al. 1993, 1994), or modulation of ongoing synaptic inhibition or facilitation (Taylor and Gandevia 2001; Lentz and Nielsen 2002). As fatigue develops and cortical stimulations are delivered during contraction, MEPs increase, demonstrating an increased excitability of motor cortical cells, a finding that is well established in the upper-limb (Taylor et al. 1996; Benwell et al. 2006; Yoon et al. 2012) and more recently the knee-extensors (Kennedy et al. 2016; Vernillo et al. 2017). Following the MEP evoked during voluntary contraction, there is a period of silence in electromyographic activity (EMG) termed the corticospinal silent period (CSP). The CSP shows an interruption in volitional drive from the cortex and withdrawal of descending input to the spinal motorneuron pool (Chen et al. 1999; Williams et al. 2014). In a fresh state, CSP duration has been attributed to periods of spinal refractoriness and cortical inhibition (up to ~ 200 ms; Chen et al. 1999; Rothwell 2009). For many years it has been thought that spinal mechanisms contribute to the initial (50–80 ms) part of the CSP (Fuhr et al. 1991; Ziemann et al. 1993). However, more recent evidence suggests that the CSP has a much longer, and more influential spinal component than previously thought (Yacyshyn et al. 2016). During fatigue there is a lengthening of CSP duration that is reflective of greater motor cortical inhibition (McKay et al. 1996; Taylor et al. 1996; Kennedy et al. 2016; Vernillo et al. 2017) mediated by receptor B of the neurotransmitter gamma-aminobutyric acid (GABA_B_; McDonnell et al. 2006).

Paired-pulse TMS protocols provide a strategy to directly evaluate the excitability of intracortical inhibitory and facilitatory networks within the motor cortex (Ni and Chen 2011). A sub-threshold TMS stimulus, which activates intracortical inhibitory circuits, reduces the size of an MEP elicited 2–5 ms later; a response termed short-interval intracortical inhibition (SICI; Kujirai et al. 1993). There is good evidence to show that this form of inhibition is caused by the activation of GABA_A_ within the primary motor cortex (Ziemann et al. 1996; Di Lazzaro et al. 2000). When SICI is measured following fatiguing exercise of the upper-limb, inhibition is reduced (Benwell et al. 2006; Maruyama et al. 2006; Takahashi et al. 2011; Hunter et al. 2016) or remains unchanged (Tergau et al. 2000). Despite the importance of knee-extensor muscles to locomotion, little is known about the response of motor cortical inhibitory networks following fatiguing lower-limb exercise. No changes in SICI have been found following heavy resistance (Thomas et al. 2017b) or intermittent (Brownstein et al. 2017; Thomas et al. 2017a) exercise. Moreover, Takahashi et al. (2011) studied the effect of fatiguing, submaximal exercise, and found this to progressively reduce corticospinal excitability and SICI when measured at rest in the knee-extensors. However, recent evidence has suggested that excitability of intracortical networks should be assessed in an active, rather than resting muscle (Gruet et al. 2013; Thomas et al. 2016a), and the response in such networks following maximal fatiguing exercise of the knee-extensors, remains to be elucidated.

It is common for investigations studying fatigue to report changes in inhibition without delineating the specific alterations in networks governed by the activity of GABA_A_ and GABA_B_. Accordingly, the aim of the present study was to investigate the effect of repeated, maximal, isometric, knee-extensor contractions on the development of fatigue, corticospinal excitability, and inhibition. It was hypothesised that the sustained, maximal contractions would elicit a substantial level of fatigue, with a concomitant reduction in corticospinal excitability and increased inhibition.

## Methods

### Participants

Thirteen, recreationally active males (age, 23 ± 2 year; stature, 1.81 ± 0.04 m; body mass, 85.2 ± 12.5 kg) volunteered to participate. Participants arrived at the laboratory in a rested state, having avoided strenuous exercise in the preceding 48 h. Volunteers also refrained from caffeine for 12 h and alcohol for 24 h prior to each trial. Prior to any experimental procedures, written informed consent was obtained from all participants and the study conformed to the latest revision of the Declaration of Helsinki. Northumbria University’s Research Ethics Committee approved all procedures.

### Experimental design

**Fig. 1:**
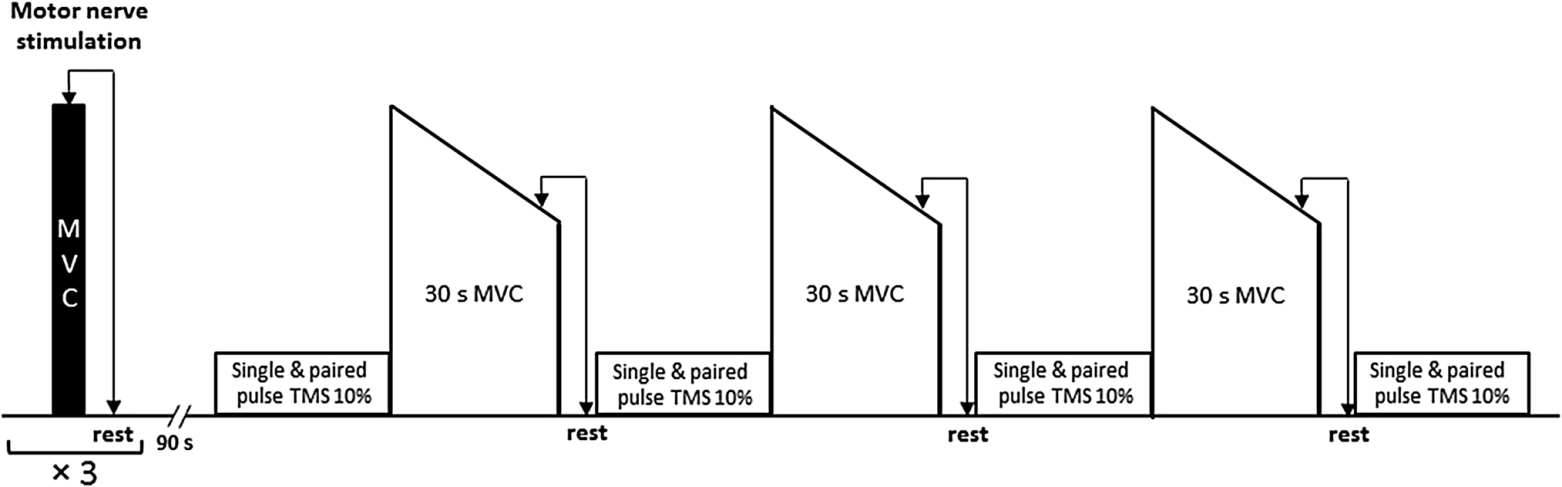
Schematic of the experimental trial. At baseline participants performed 3 knee-extensor maximum voluntary contractions (MVCs) with motor nerve stimulation delivered to the femoral nerve during and 2 s post, to determine voluntary activation and potentiated twitch force, respectively. Single and paired responses (6 of each) to motor cortical stimulation were then elicited during a 10% MVC. Thereafter, participants performed a sustained (30 s) maximal contraction with femoral nerve stimulation delivered during the final 5 s. An additional, stimulation was delivered at rest immediately after the 30 s contraction, participants then contracted at 10% of the final preceding force for 1 min whilst single and paired (6 of each) motor cortical stimuli were delivered. The procedure was repeated 3 times

Participants visited the laboratory on two occasions, firstly for habituation to the measurement tools and procedures, then, secondly for the experimental trial. During the experimental visit participants performed three maximal, isometric voluntary contractions (MVC1, MVC2, MVC3) with the dominant knee-extensors, each sustained for 30 s and separated by 60 s. Following each contraction neuromuscular function, corticospinal excitability, CSP and SICI were assessed. Each visit was separated by at least 3 days; a schematic of the experimental procedure is provided in Fig. 1.

### Force and EMG recordings

During all voluntary and evoked contractions, knee-extensor force (N) was measured using a calibrated load cell (MuscleLab force sensor 300, Ergotest Technology, Norway). The load cell was fixed to a custom-built chair and connected to a non-compliant cuff attached around the participant’s dominant leg, superior to the malleoli. Participants sat upright in the chair with their hips and knees at 90° of flexion, they were instructed to grasp the handles on the side of the chair for support during contractions. EMG activity was recorded from the rectus femoris (*RF*), vastus lateralis (*VL*) and vastus medialis (*VM*). Surface Ag/AgCl electrodes (Kendall H87PG/F, Covidien, Mansfield, MA, USA) were placed 2 cm apart over the muscle bellies and a reference electrode was placed over the patella. Electrode placement was marked with permanent ink to ensure a consistent placement between both sessions. The electrodes were used to record the compound muscle action potential (M-wave) elicited by electrical stimulation of the femoral nerve and MEPs elicited by TMS. The root mean square EMG (rmsEMG) activity was measured during the first and last 5 s of each sustained MVC and the pre-stimulus rmsEMG (80 ms) activity was measured during all 10% MVC contractions. All rmsEMG values were normalised to the maximal M-wave at the respective time point (rmsEMG/*M*_max_). Signals were amplified (gain × 1000 for EMG; ×300 for force, CED 1902, Cambridge Electronic Design, UK), band-pass filtered (EMG only: 20–2000 Hz), digitised (4 kHz; CED 1401, Cambridge Electronic Design, UK), acquired and analysed off line (Spike2 v7.12, Cambridge Electronic Design, UK).

### Femoral nerve stimulation

Single, electrical stimuli (200 µs pulse width) were delivered to the femoral nerve through surface electrodes (CF3200, Nidd Valley Medical Ltd, North Yorkshire, UK) using a constant-current stimulator (DS7AH, Digitimer Ltd, Welwyn Garden City, Hertfordshire, UK). In line with previous investigations from our laboratory (Goodall et al. 2015; Thomas et al. 2016b, 2017b), the cathode was positioned over the nerve, high in the femoral triangle, whilst the anode was placed midway between the greater trochanter and the iliac crest. Single stimuli were delivered to the relaxed muscle beginning at 40 mA, the intensity was increased by 20 mA until a plateau occurred in twitch amplitude and M-wave (*M*_max_). Supramaximal stimulation was delivered by increasing the final stimulator output by 30% (mean current, 208 ± 57 mA). The positions of the stimulating electrodes were marked with indelible ink to ensure consistent placement during each session. At each time point muscle contractility was assessed for the peripherally derived resting twitches as twitch amplitude (*Q*_tw,pot_: maximum twitch tension) and membrane excitability was inferred from the peak-to-peak amplitude of the electrically evoked *M*_max_.

### Transcranial magnetic stimulation

Single- and paired-pulse TMS were delivered using a concave double cone coil (110 mm diameter; maximum output 1.4 T), powered by a BiStim unit and two Magstim 200^2^ stimulators (The Magstim Company Ltd, Whitland, UK). The coil was held over the vertex to stimulate the contralateral hemisphere to the dominant leg (induced current = postero-anterior). The optimal position to elicit a large MEP in the knee-extensors was identified during a brief 50% MVC contraction, with the stimulator output set at 50%. The optimal position was marked on the scalp with indelible ink to ensure a reproducible site of stimulation. Active motor threshold (aMT) was determined during a 10% MVC as the minimum stimulus intensity that elicited a consistent MEP in the *RF*, of at least 200 *µ*V in three of five stimulations (mean stimulator output, 44 ± 5%).

### Assessment of neuromuscular function

At baseline, MVC force was determined from three maximal 3 s contractions. Femoral nerve stimulation was delivered during each of these contractions and an additional stimulus was delivered at rest, ~ 2 s after, to determine voluntary activation (VA; Merton 1954) and the *Q*_tw,pot_, respectively. During the 30-s sustained contractions, MVC was taken as the maximal force achieved in the first 5 s and femoral nerve stimulation was delivered during the final 5 s. At the end of each MVC, participants were instructed to completely relax and an additional femoral nerve stimulus was delivered to determine *Q*_tw,pot_ and subsequently, VA. Following the resting stimulus, participants were instructed to contract at 10% of the preceding final MVC force for the assessment of corticospinal excitability, CSP, and SICI (see below). Once the 60-s assessment period had elapsed, participants began the next sustained maximal contraction and this procedure was repeated until 3 sustained MVCs had been completed. Strong verbal encouragement was provided throughout each sustained contraction.

### Corticospinal excitability, silent period, and short-interval intracortical inhibition

During each neuromuscular assessment, six single and six paired-pulse magnetic stimuli were delivered in an alternate order, the mean responses were used to quantify corticospinal excitability and inhibition, respectively. Stimuli were separated by 4–6 s and delivered whilst participants held an isometric knee-extensor contraction at 10% of the final MVC force. Following each unconditioned MEP, the duration of the CSP was determined as the interval from stimulation to the time at which post-stimulus EMG had resumed (Goodall et al. 2010). Provisional data from our laboratory which investigated different contraction strengths, ISIs, and stimulation intensities to produce the greatest SICI response dictated the method used. To elicit SICI, a sub-threshold conditioning stimulus (0.7 × aMT) was followed by a supra-threshold (1.2 × aMT) unconditioned test stimulus using an inter-stimulus interval (ISI) of 2 ms. The SICI ratio was determined by comparing the amplitude of the conditioned and unconditioned responses. The ratio between the unconditioned MEP and resting *M*_max_ was used for the quantification of corticospinal excitability at each respective time point. During all contractions, visual feedback of the target force was provided via a computer monitor.

### Data analysis

Voluntary activation measured through stimulation of the femoral nerve, was quantified using the twitch interpolation technique (Merton 1954). Voluntary activation was quantified by comparing the amplitude of the SIT during MVCs with the amplitude of the resting *Q*_tw,pot_ elicited 2 s post-MVC: VA (%) = (1 − [SIT/*Q*_tw,pot_] × 100). The peak-to-peak amplitudes of evoked MEPs and *M*_max_ were calculated offline.

### Statistical analysis

Data are presented as means ± SD throughout. A one-way repeated measures analysis of variance (ANOVA) was used to assess changes in all outcome measures. Assumptions of sphericity were explored and controlled for all variables using the Greenhouse–Geisser adjustment, where appropriate. Where significant main effects were detected, and pairwise comparisons between time-points are reported, the Tukey method was used to make adjustments for multiple comparisons (Graphpad Prism, v5.04, La Jolla, CA, USA). Statistical significance was assumed at *P* ≤ 0.05.

## Results

### Neuromuscular function

**Fig. 2:**
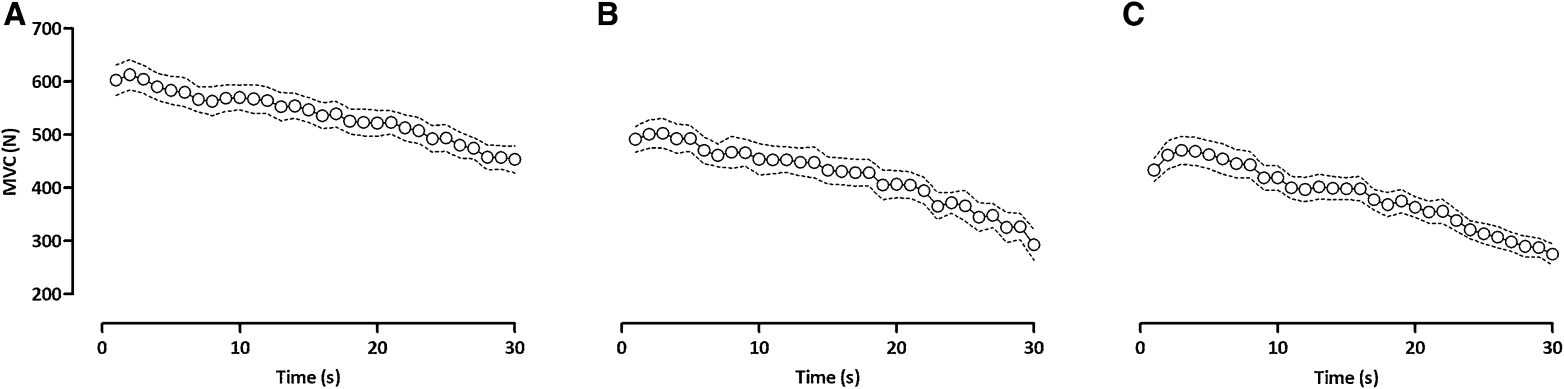
Average (1 s) knee-extensor force throughout the three sustained, maximal contractions (**A** MVC1; **B** MVC2; **C** MVC3). Values are means ± SD (dashed lines)

**Fig. 3:**
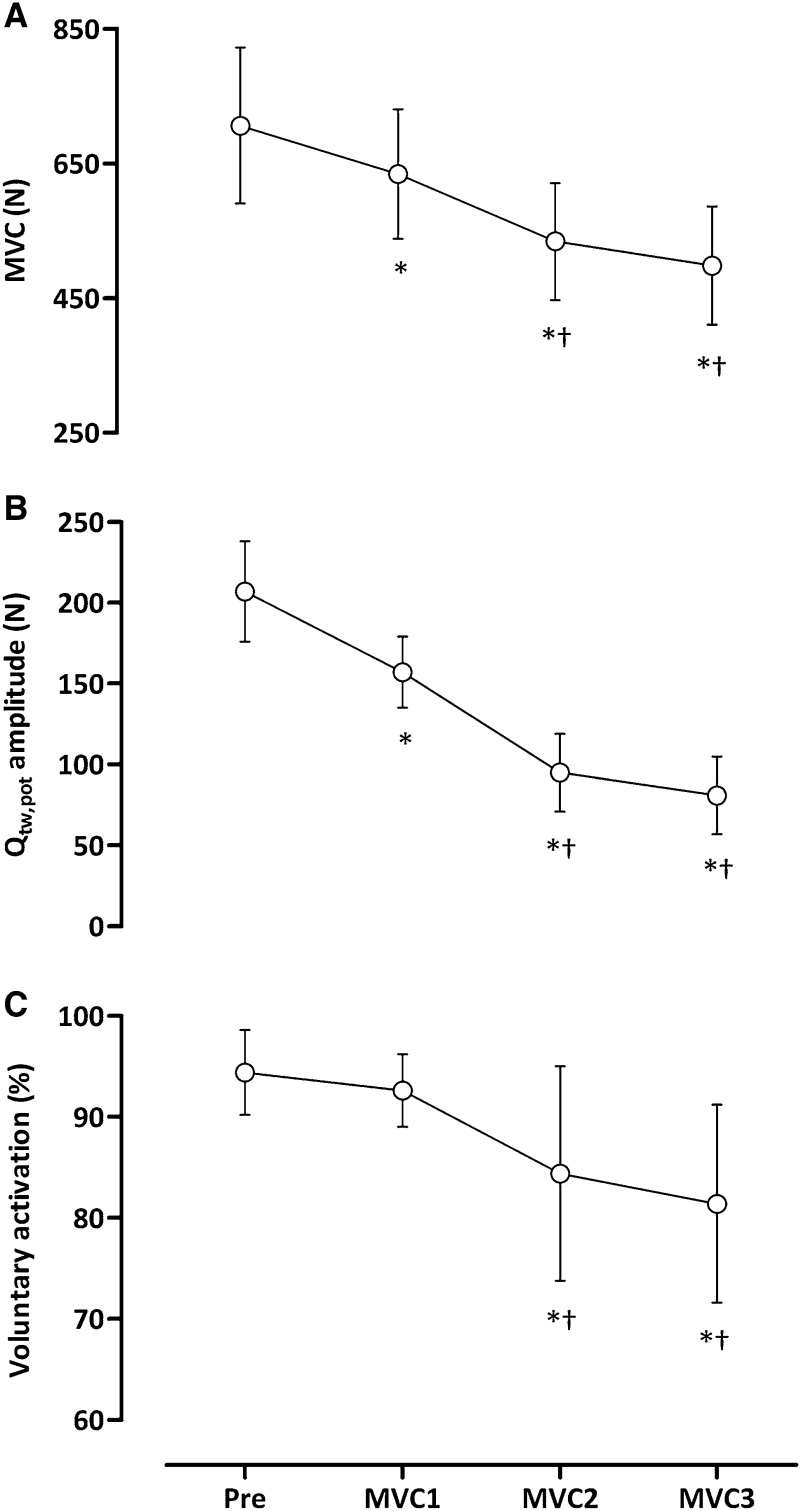
Maximum voluntary contraction (**A**), potentiated knee-extensor twitch force (**B**) and voluntary activation measured with motor nerve stimulation (**C**) at baseline, and following three sustained, maximal contractions. *P < 0.05 vs. baseline, ^†^ *P* < 0.05 vs. MVC1. Values are means ± SD

Force reduced throughout the protocol (*F*_3,36_ = 52.83, *P* < 0.0001; Fig. 2), with peak values during MVC1 (− 10 ± 7%, *P* < 0.01), MVC2 (− 24 ± 9%, *P* < 0.001) and MVC3 (− 29 ± 19%, *P* < 0.001) decreased compared to baseline (706 ± 115 N). The reductions in maximal force during MVC2 and MVC3 were greater than MVC1 (*P* < 0.001) (Fig. 3A). The reduced MVC was accompanied by a significant reduction in *Q*_tw,pot_ (*F*_3,36_ = 135.60, *P* < 0.0001) indicative of peripheral fatigue (Fig. 3B). The decline in *Q*_tw,pot_ amplitude from baseline (207 ± 30 N) was evident following MVC1 (− 23 ± 10%, *P* < 0.001) with further reductions following MVC2 (− 53 ± 13%, *P* < 0.001) and MVC3 (− 60 ± 13%, *P* < 0.001). The reductions in *Q*_tw,pot_ following MVC2 and MVC3 were greater than MVC1 (*P* < 0.001). Central fatigue was evident via reductions in VA throughout the protocol (*F*_3,36_ = 9.14, *P* < 0.0001). In comparison to baseline (94 ± 4%), VA was reduced following MVC2 (− 10 ± 13%, *P* < 0.01) and MVC3 (− 13 ± 10%, *P* < 0.001). The reduction in VA during MVC2 and MVC3 was greater than MVC1 (*P* < 0.05; Fig. 3C).

### Corticospinal excitability, CSP and SICI

**Fig. 4:**
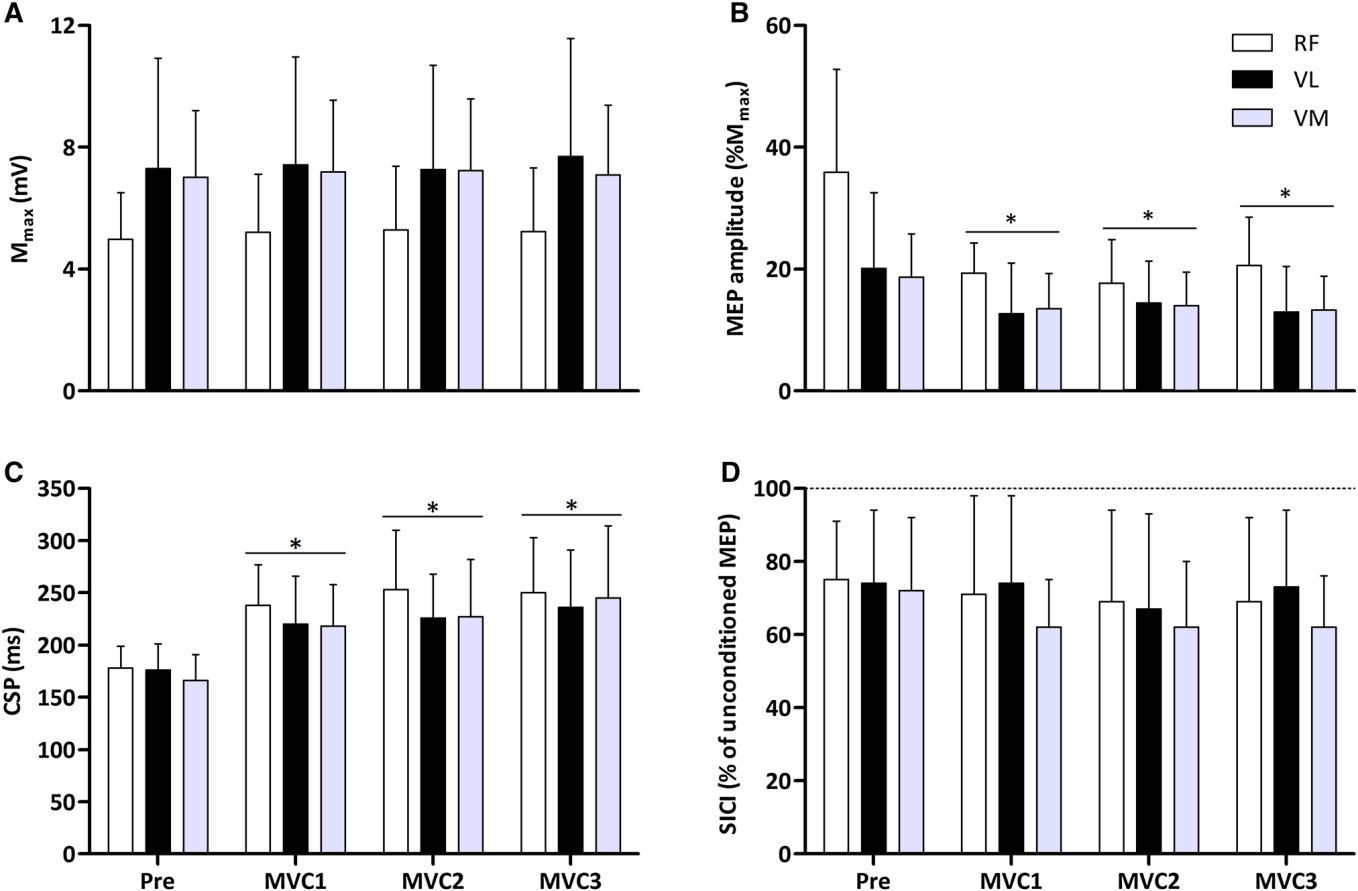
The maximal M-wave (*M*_max_; **A**), MEP amplitude (%*M*_max_; **B**), the corticospinal silent period (CSP; **C**) and short-interval intracortical inhibition (SICI) determined during a 10% MVC contraction (**D**) at baseline, and following three sustained, maximal knee-extensor contractions. *P < 0.05 vs. baseline. Values are means ± SD

**Fig. 5:**
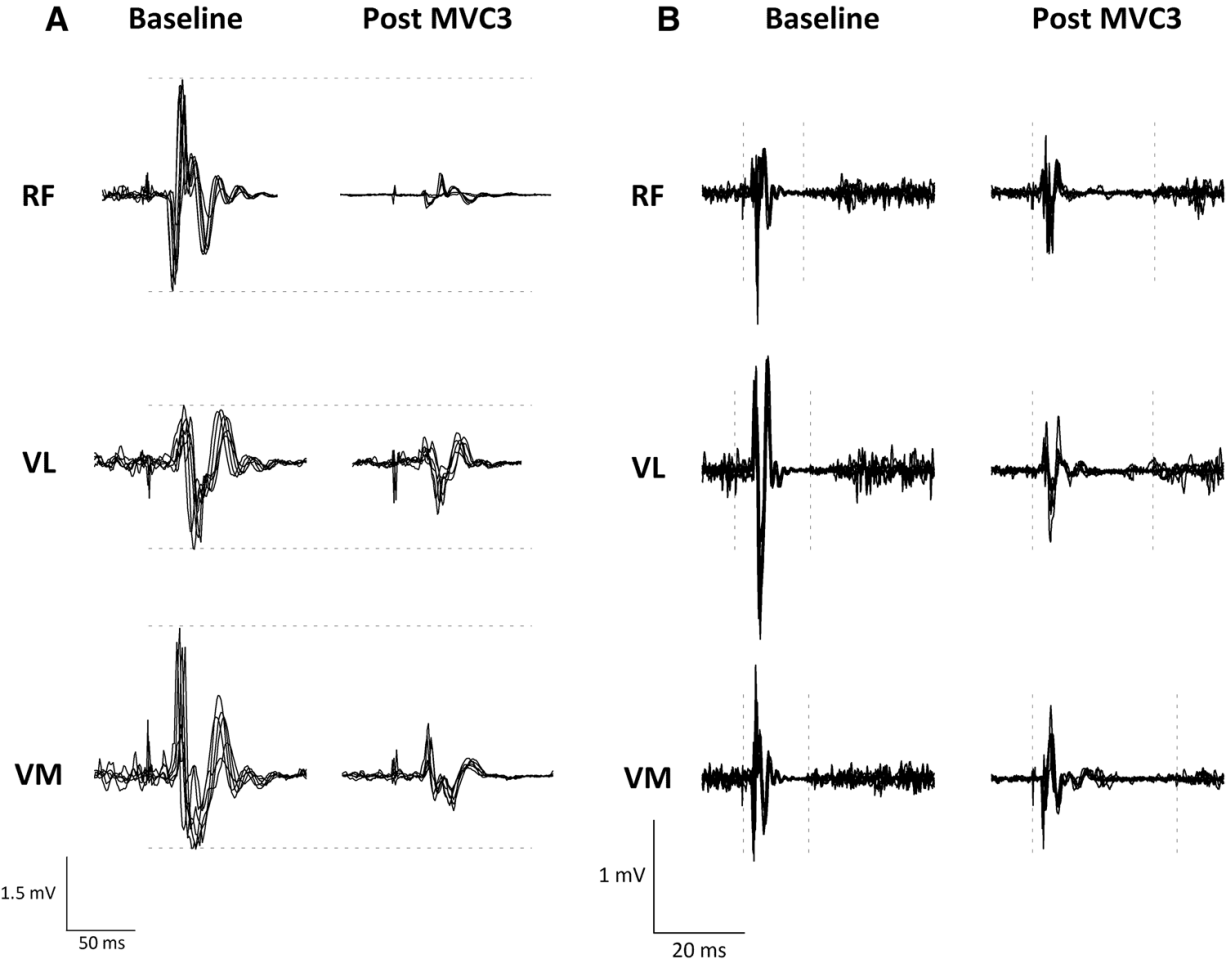
**A** shows the MEP response in each knee-extensor muscle, the horizontal dashed line demonstrates the reduction in amplitude in comparison to baseline, following the 3rd sustained MVC. **B** shows the corticospinal silent period measured in each knee-extensor muscle (see “Methods” for quantification). The vertical dashed lines are placed at the time of stimulation and at the end of the silent period, the traces on the right show increased silent period durations following the 3rd sustained MVC. For each panel, respectively, data are from a representative participant and the responses to six unconditioned motor cortex stimuli are overlaid. *RF* rectus femoris, *VL* vastus lateralis, *VM* vastus medialis

There were no changes in *M*_max_ amplitude for any knee-extensor muscle (*F*_3,36_ ≤ 0.60, *P* ≥ 0.619; Fig. 4A); however, MEP amplitudes were reduced throughout the protocol (*F*_3,36_ ≥ 2.89, *P* ≤ 0.015). Consequently, corticospinal excitability (MEP/*M*_max_) was reduced in the knee-extensors following MVC1 (Fig. 4B). CSP duration was similar at baseline in all knee-extensor muscles (*RF*, 178 ± 21 ms; *VL*, 176 ± 25 ms; *VM*, 166 ± 25 ms) and was significantly lengthened following MVC1 (*RF*, 34 ± 14%; *VL*, 25 ± 21%; *VM*, 32 ± 18%; *F*_3,36_ ≥ 20.90, *P* < 0.001). CSP duration progressively increased following MVC3 in the *VM* (*P* = 0.022 vs. MVC1; *P* = 0.050 vs. MVC2) and *VL* (*P* = 0.015 vs. MVC1) (Fig. 4C). Baseline SICI was similar in all knee-extensor muscles (*RF*, 0.75 ± 0.17; *VL*, 0.70 ± 0.17; *VM*, 0.70 ± 0.18) and did not change at any point during the protocol (*F*_3,36_ ≤ 1.34, *P* ≥ 0.276; Fig. 4D). The changes in MEP amplitude and CSP duration are shown for a representative participant in Fig. 5A, B, respectively. During each assessment period, there were time effects for the unconditioned *RF*, *VL* and *VM* MEP amplitudes and the conditioned *VL* and *VM* MEP amplitudes; these responses are shown in Supplementary Fig. 1.

### rmsEMG activity

The rmsEMG/*M*_max_ was reduced in the *VL* (*F*_5,31_ = 3.94, *P* = 0.004) during the sustained MVCs, but no changes were observed in the *RF* (*F*_2,28_ = 2.72, *P* = 0.075) or *VM* (*F*_2,23_ = 3.02, *P* = 0.072) (Supplementary Fig. 2). The pre-stimulus rmsEMG/*M*_max_ during the assessment sets between sustained contractions was similar for all knee-extensor muscles and did not change after any sustained MVC (*P* ≥ 0.119). Furthermore, there were no time effects for the pre-stimulus rmsEMG measured during the 60-s assessment periods between each sustained contraction (Supplementary Fig. 3).

## Discussion

This study determined whether the development of fatigue following repeated, isometric, maximal contractions performed with the knee-extensors, altered corticospinal excitability and inhibition. The novel findings of this study are that (1) the fatigue elicited was concurrent with a reduced corticospinal excitability and increased CSP duration, whereas (2) SICI did not change from baseline at any point throughout the protocol. These data demonstrate that the fatigue elicited by repeated, sustained contractions, caused impairments in neuromuscular function coupled with differential responses in excitability and inhibitory networks within the motor cortex. Specifically, activity of inhibitory networks mediated by GABA_B_ were increased with fatigue, however, those mediated by GABA_A_ were not.

### Mechanisms of neuromuscular fatigue

The sustained maximal contractions elicited progressive neuromuscular fatigue that was accompanied by reductions in maximal force generating capacity (Fig. 3A), resting potentiated twitch force (Fig. 3B) and voluntary activation (Fig. 3C). The final force attained in the third MVC was reduced by ~ 40% compared to baseline; a value that is less than other investigations studying responses in the knee-extensors following a 2-min sustained contraction, where a reduction of ~ 75% was observed (Goodall et al. 2009; Place et al. 2009; Kennedy et al. 2016; Vernillo et al. 2017). This is likely because of the shorter total contraction time in the present study, coupled with the minute intervening contractions at 10% MVC, which would have allowed for greater restoration of blood flow than a sustained 2-min contraction. A restoration of blood flow enables the recovery of muscle force following exercise-induced fatigue (Kennedy et al. 2016), and might explain why a higher force was observed at the start of each contraction in comparison to the value at the end of the preceding contraction (Fig. 2). The reductions in MVC were accompanied by progressive declines in the *Q*_tw,pot_, indicative of peripheral muscle fatigue (Fig. 3B). The decline in the *Q*_tw,pot_ was greatest following MVC3 (− 60%), which is similar to that observed after sustained (Vernillo et al. 2017) and dynamic (Rossman et al. 2012) knee-extensor exercise, but is more than what is observed following fatiguing locomotor exercise (Amann et al. 2007; Sidhu et al. 2009; Goodall et al. 2012, 2017; Thomas et al. 2016b; Ansdell et al. 2017). As with most investigations that demonstrate peripheral fatigue (Goodall et al. 2010; Rossman et al. 2012; Temesi et al. 2015; Thomas et al. 2016b), the maximal M-wave elicited by supramaximal femoral nerve stimulation was unchanged throughout the experiment. Thus, the peripheral fatigue elicited was likely related to postsynaptic disturbances in the excitation–contraction coupling process. Specifically, impairments to intracellular Ca^+2^ regulation in the sarcoplasmic reticulum might reduce Ca^+2^ sensitivity, leading to a reduction in mechanical output and therefore, muscle fatigue (MacIntosh et al. 2012).

There was also a contribution from central mechanisms of fatigue as evidenced by the reduced voluntary activation from MVC2 (Fig. 3C), that is, despite maximal effort, there was insufficient drive to motor units to generate maximal force (Gandevia 2001). During a 2-min maximal contraction of the biceps, Schillings et al. (2003) suggested that mechanisms of peripheral and central fatigue do not change in parallel. Similar to the present study, after approximately 1 min, the contribution of peripheral fatigue tended to level off, and the further decrease in voluntary force can be attributed to mechanisms of central fatigue (Schillings et al. 2003). During the initial part of a sustained contraction the output of muscle is highest, combined with a high metabolic demand and occluded blood flow. Throughout the second half of the contraction, the continuous and repetitive firing of motoneurones would be difficult to maintain, ultimately leading to further decrements in force production (Schillings et al. 2003, 2005; Taylor and Gandevia 2008). Although not measured in the present study, it is likely that that supraspinal mechanisms of fatigue would have contributed to the force loss as the contractions progressed (Gruet et al. 2014). Collectively, these results demonstrate that peripheral fatigue manifests early during the repeated, maximal, sustained contractions; however, with time, there are greater central contributions to the exercise-induced fatigue.

### Neuromuscular fatigue and corticospinal excitability

Since fatigue was observed, the present study was able to investigate the associated changes in corticospinal function with fatigue. Following MVC1, corticospinal excitability was reduced (Fig. 4A) and despite progressive neuromuscular fatigue evident following MVC2 and MVC3, further reductions in corticospinal excitability were not observed. The reduction in corticospinal excitability is opposite to what has recently been reported for the knee-extensors (Kennedy et al. 2016; Vernillo et al. 2017), yet these data are unsurprising because both investigations elicited the MEPs during a sustained contraction. Indeed, this increase in MEP during the fatiguing contraction suggests an increase in the excitability of motor cortical cells (McKay et al. 1996; Taylor et al. 1996; Hilty et al. 2011); however, shortly after termination of a sustained contraction, MEPs either return to baseline (Kennedy et al. 2016) or reduce (Kotan et al. 2015). Some of the proposed mechanisms for the depression in corticospinal excitability include changes in membrane properties of corticospinal neurons and changes in the efficiency of excitatory synaptic inputs onto corticospinal neurons that occur as a result of high levels of activity during the fatiguing contraction (Brasil-Neto et al. 1993; McKay et al. 1996; Samii et al. 1996; Taylor et al. 1996; Kotan et al. 2015). A change in MEP size, however, does not solely reflect changes at the motor cortex, as these potentials can also be affected by changes in the motoneurones and muscle fibres (Taylor and Gandevia 2004; Taylor 2006; McNeil et al. 2009). Furthermore, corticospinal excitability has also been shown to reduce in response to a period of constant electrical stimulation of the median nerve (Kotan et al. 2015), demonstrating a role for afferent feedback. It is well known that Group III and IV muscle afferents fire during continuous muscle contraction in response to mechanical and metabolic changes during skeletal muscle contraction (Taylor and Gandevia 2008), and previous work has shown such afferent feedback to reduce corticospinal excitability (Ridding and Rothwell 1997; Ziemann et al. 1998). Despite these findings, a recent investigation has found excitability of the *VL* motoneurone pool to be unaffected by feedback from Group III and IV muscle afferents (Kennedy et al. 2016). Nevertheless, high levels of cortical activity during the sustained contractions, along with a heightened afferent feedback, are plausible explanations for the reduced corticospinal excitability observed in the present study.

### Neuromuscular fatigue and motor cortical inhibition

The diminished corticospinal excitability in all knee-extensor muscles occurred in parallel with increased inhibition, in the form of lengthened silent periods (Figs. 4B, 5B). CSP duration was increased compared to baseline in all knee-extensor muscles following all MVCs. As the protocol ensued, progressive increases in CSP duration were observed (*VM* and *VL*) signifying an augmented level of inhibition. For many years it has been thought that spinal mechanisms contribute to the initial (50–80 ms) part of the CSP (Fuhr et al. 1991; Ziemann et al. 1993). However, more recent evidence suggests that the CSP has a much longer, and more influential spinal component than previously thought (Yacyshyn et al. 2016). The lengthening of CSP duration when fatigue is experienced, has been attributed to increased cortical inhibition only (Kennedy et al. 2016). Kennedy et al. (2016) have recently shown unchanged motoneuronal responsiveness, but increased CSP duration, following fatiguing isometric exercise performed with the knee-extensors. These data, coupled with the present study, suggest that fatigue-related afferents influence inhibition mediated by GABA_B_ receptors within the motor cortex. These data confirm previous observations from blockade studies showing that when feedback from such afferents is blocked, by the injection of intrathecal fentanyl, CSP duration does not increase (Hilty et al. 2011; Sidhu et al. 2017). Conversely, SICI, which tests the excitability of GABA_A_-mediated intracortical inhibitory networks within the motor cortex was unaffected by fatigue. At baseline, SICI measured in the active knee-extensors, was similar to that previously reported (~ 0.75; Brownstein et al. 2017; Thomas et al. 2017b). However, in contrast to the reduction in corticospinal excitability following MVC1, SICI was unaltered following any of the sustained contractions (Fig. 4C); which concurs with some literature (Tergau et al. 2000; Brownstein et al. 2017; Thomas et al. 2017a), but not others, who report reduced inhibition (Benwell et al. 2006; Maruyama et al. 2006; Takahashi et al. 2011; Hunter et al. 2016).

Our data suggest that GABA_B_ receptors play an important role in modulating the intracortical inhibitory response within the motor cortex during fatiguing exercise. The effect of GABA released by the presynaptic axon terminals of inhibitory interneurons depends on the receptor on the postsynaptic cell membrane it interacts with. McCormick (1992) explained how GABA_A_ receptors are mediated by a short-lasting, Cl^−^-dependant, component of stimulation-induced inhibitory postsynaptic potentials; whereas, a long-lasting K^+^-dependent component, results from activation of postsynaptic GABA_B_ receptors. The latter component is only evident when inhibitory interneurons are strongly activated; suggesting that activation of GABA_B_ receptors requires a higher GABA concentration or a longer exposure to GABA than that necessary for the activation of GABA_A_ receptors (Otis and Mody 1992). Thus, the fatigue elicited by the repeated, sustained contractions, was likely in parallel with a substantial release of GABA from inhibitory interneurons. Such a release of GABA is not easily resolved in the synaptic cleft, thus favouring GABA_B_ receptor activation (Thompson and Gahwiler 1992), explaining the prolonged CSP but unchanged SICI.

The conflicting results within the literature for the response in SICI during fatigue are possibly due to subtle differences in the way in which SICI is assessed. For example, SICI will depend on whether the muscle is in an active or resting state, whether the unconditioned MEP size is of a specific amplitude rather than being based on 1.2 × aMT, by varying the strength of sub-threshold conditioning stimulations, and altering the inter-stimulus interval. Many of these variables can be manipulated (Ortu et al. 2008) and it is important to optimise the methods of assessing SICI in a particular muscle prior to studying a response. Provisional data from our laboratory which investigated different contraction strengths, ISIs, and stimulation intensities to produce the greatest SICI response dictated the method used in the present study. Rather than basing the test pulse on a specific MEP size, we use 1.2 × aMT, with the aMT determined during a 10% MVC. Indeed, with changes in corticospinal excitability following MVC1, thresholds may have also changed. However, it was not possible to adjust the stimulator output to achieve a specific MEP size, or to re-establish thresholds during the 60-s assessment period between the sustained contractions. Rather, we elicited MEP and SICI measurements with a consistent level of mechanical output (relative 10% MVC) at all-time points. It was important that we controlled for the level of mechanical output because assessing SICI at the absolute 10% MVC level would mean the contraction strength would have been higher when the muscle was in a fatigued state. Indeed, the minute between the sustained contractions would had presumably allowed for some recovery in force, such that, towards the end of the 60-s period the contraction held might have been more akin to around 5, rather than 10% MVC. The time effects observed for MEP and SICI amplitudes throughout these assessment periods can be explained by the first 1 or 2 responses following each sustained MVC, being larger than the following 4 or 5 responses (see Supplementary Fig. 1). There were no changes in the *M*_max_ at these time points and the larger MEP responses immediately post the sustained MVCs are not deemed to be ‘facilitated’, because they are no more than the baseline value. Similar findings, elicited in resting muscle, have previously been reported and termed central postactivation facilitation, which is related to the balance of neurotransmitters during fatigue (Brasil-Neto et al. 1993, 1994). For these increased, isolated responses, a greater level of inhibition is observed (reduced SICI ratio); however, this was not representative of the mean response during each assessment period (Fig. 4D). Thus, we do not believe this finding changes our conclusions as the responses to TMS should not be observed in isolation, it is far more prudent to study the mean of a set of responses as is done in the present study. Moreover, the evoked response during each assessment period were elicited with the same level of pre-stimulus EMG (Supplementary Fig. 3), thus, we do not believe the effect of ‘recovery’ throughout the 60-s assessment period impacts the conclusions made in the present study.

## Conclusion

In summary, our findings indicate that a progressive level of neuromuscular fatigue within the knee-extensors coincides with a reduced corticospinal excitability and increased CSP duration, demonstrating modulation of specific neural networks within the corticospinal pathway. Additionally, our data reveal changes in the behaviour of networks within the motor cortex with fatigue and future work should aim to understand the mechanisms for the differential responses in inhibitory networks mediated by GABA receptors.

## Electronic supplementary material

Below is the link to the electronic supplementary material.

Supplementary Figure 1. Raw single and conditioned MEP amplitudes in the *RF* (A), *VL* (B) and *VM* (C) elicited during a 10% MVC contraction at baseline and following each sustained MVC. The 10% MVC contraction intensity was based on the final force achieved in the preceding sustained MVC (TIF 2842 KB)

Supplementary Figure 2. Knee-extensor rmsEMG/*M*_max_ measured from the *RF* (A), *VL* (B), and *VM* (C) during the first, and last, 5 s of the sustained contractions. ** significant time effect (TIF 2724 KB)

Supplementary Figure 3. Pre-stimulus (80 ms) knee-extensor rmsEMG/*M*_max_ measured from the *RF* (A), *VL* (B) and *VM* (C) throughout the 60 s assessment period at baseline, and following each sustained MVC (TIF 2737 KB)
